# Macaque monkeys learn and perform a non-match-to-goal task using an automated home cage training procedure

**DOI:** 10.1038/s41598-021-82021-w

**Published:** 2021-01-29

**Authors:** Stefano Sacchetti, Francesco Ceccarelli, Lorenzo Ferrucci, Danilo Benozzo, Emiliano Brunamonti, Simon Nougaret, Aldo Genovesio

**Affiliations:** 1 Department of Physiology and Pharmacology, SAPIENZA, University of Rome, Piazzale Aldo Moro 5, 00185 Rome, Italy; 2 PhD Program in Behavioral Neuroscience, Sapienza University of Rome, Rome, Italy

**Keywords:** Operant learning, Working memory

## Abstract

In neurophysiology, nonhuman primates represent an important model for studying the brain. Typically, monkeys are moved from their home cage to an experimental room daily, where they sit in a primate chair and interact with electronic devices. Refining this procedure would make the researchers’ work easier and improve the animals’ welfare. To address this issue, we used home-cage training to train two macaque monkeys in a non-match-to-goal task, where each trial required a switch from the choice made in the previous trial to obtain a reward. The monkeys were tested in two versions of the task, one in which they acted as the agent in every trial and one in which some trials were completed by a “ghost agent”. We evaluated their involvement in terms of their performance and their interaction with the apparatus. Both monkeys were able to maintain a constant involvement in the task with good, stable performance within sessions in both versions of the task. Our study confirms the feasibility of home-cage training and demonstrates that even with challenging tasks, monkeys can complete a large number of trials at a high performance level, which is a prerequisite for electrophysiological studies of monkey behavior.

## Introduction

In recent decades, a large amount of data has been collected from animal studies done in zoos. These data come from tasks or observations made directly in the home cage of the animals, using various setup designs^1^. Many of these studies investigated the cognition of nonhuman primates (NHPs) and were conducted on the monkeys within their social group^2,3^. A recent survey that considered publications from 2000 to 2015 on topics specific to NHPs such as “social group” and “cognition” found that studying monkeys in their social group can be beneficial for their well-being^4^. This systematic literature survey showed that the most common environment in which these studies are conducted are field sites, where a mobile setup is used to test macaque monkeys directly in their environment^5^. In parallel, neurophysiologists working with NHPs started conducting experiments directly in the monkey’s home cage, using standardized training protocols^6–10^.

In neurophysiology, NHPs represent a valid experimental model because of the homology of most of their brain regions with those of humans, and their cognitive and social abilities can be studied using complex visuomotor tasks in a laboratory setup^11–16^. However, for this purpose, once training starts, they must be moved daily from their home cage to a special primate chair and carried to an experimental room where they perform the task. Even if protocols that allow monkeys to become accustomed to positive-reinforcement training can reduce the time spent by researchers in the initial stages of training^17,18^, the relocation procedure is nevertheless time-consuming and increases the risk of bites, scratches, and escape by the monkey. In recent years, increasing attention has been focused on the welfare of laboratory animals, and researchers constantly refine their methods to minimize the animals’ stress and improve their well-being^19–23^, in accordance with the principles of the 3Rs (Replacement, Reduction, and Refinement)^24^.

Previous studies have provided evidence of the ability of animals such as rodents^25^ and NHPs other than macaque monkeys to perform cognitive tasks in their home cage^19,26–31^. The same paradigm was also used with macaque monkeys, and it proved to be an excellent tool for enrichment^32–34^ and for training animals in cognitive tasks^26,28,35–37^. Some of these studies required the use of a joystick to complete the required task^7,9,10^, were intended to teach complex discrimination tasks^38^, or investigated constructs such as metacognition^39^, cognitive flexibility^40^, and working memory^41–43^. However, to our knowledge, none of these studies investigated the ability of NHPs in their home cage to perform a task that requires continuous involvement throughout the session because each trial depends on the previous one.

Since the home cage is a context in which the monkeys are free to move around and to direct their attention elsewhere, without any constraint forcing an interaction with the setup, we wanted to evaluate the ability of the monkeys to keep their attention focused on the task. For this purpose, we performed two experiments to test both the feasibility and the possible limitations of home-cage training. We adapted tools previously described^33,34^ to our experimental needs. These adaptations concerned both the design of the setup and the protocol used to train the monkeys to perform the cognitive tasks. We evaluated their ability to maintain continuous involvement with the setup and constant engagement in the task. This initial training will also be used in neural recordings, which we plan to carry out at a later stage, using the traditional primate-chair approach with the same tasks. In the first experiment, we tested whether a cognitively demanding visuomotor task, the non-match-to-goal (NMTG) task, could be learned by the monkeys directly in their home cage, and how this learning process occurred. The task is considered cognitively demanding because, unlike other tasks used in electrophysiology, the monkeys are required to take into account information from the previous trial to make the correct choice.

In the second experiment, based on recent studies revealing the ability of macaque monkeys to learn in the so-called “ghost display” condition^44,45^, we tested the monkeys’ ability to perform a second version of the same task that required interacting in turns with a “ghost agent”. The trials performed by the monkey were interspersed with trials performed by the ghost agent; therefore, in all the trials performed by the monkey after the ghost agent, the correct choice depended on the choice made by the ghost agent.

Since the home cage is a context that allows the monkeys to move freely without limitation, we wanted to test if the training could be effective in such an environment for both experiments. Thus, we evaluated the stability of the monkeys’ involvement in the task, that is, whether their task execution was continuous or sporadic, and the stability of their performance within sessions. Our results reveal that the monkeys quickly learned to interact with the home-cage setup and the task rule, taking into account their previous choices and those of the ghost agent, and exhibited stable performance within the sessions, both during the phases of constant interaction with the apparatus and in the final stages of the sessions, in which the interaction became more sporadic.

## Experimental procedures

### General

#### Animals

For this study, we used two male macaque monkeys (*Macaca mulatta*), monkey L and monkey N (both 12 years old) and both monkeys weighed about 12 kg during the experiment. They had been caged together since they arrived at the animal facility at the age of six. Neither monkeys was naïve to the typical experimental setup. At their arrival at the facility, they were both trained for a short time to sit in a primate chair, interact with a touchscreen, and perform a visuomotor task involving an arm movement for a total of 116 (monkey L) and 28 (monkey N) sessions. The instrumental task consisted of choosing between gray squares displayed in two different positions on the touchscreen. The correct target changed position from one trial to another and the monkeys had to alternate their choices between right and left positions to be rewarded. Subsequently, neither monkeys received any further training or experience with an experimental apparatus of any kind until the training carried out in this study in their home cage.

The home cage was composed of four sections with sliding doors between them, and was equipped with a guillotine door at the front. The light cycle was centrally controlled and set to a 12/12 h dark/light cycle (light on between 06:00 am and 06:00 pm). The monkeys had free access to food for the duration of the experiment (Altromin A 6024 pellets; Altromin Spezialfutter, Lage, Germany).

The monkeys were tested for 5 months. During this testing period they received fresh fruit and vegetables and 200 ml of water per day on average, in addition to the water they received during the sessions. The additional water was given through a drinking bottle attached to the cage. Housing conditions, animal care, and experimental procedures conformed to the European (Directive 2010/63/EU) and Italian (DD.LL. 116/92 and 26/14) laws on the use of NHPs in scientific research.

#### Apparatus

**Figure 1:**
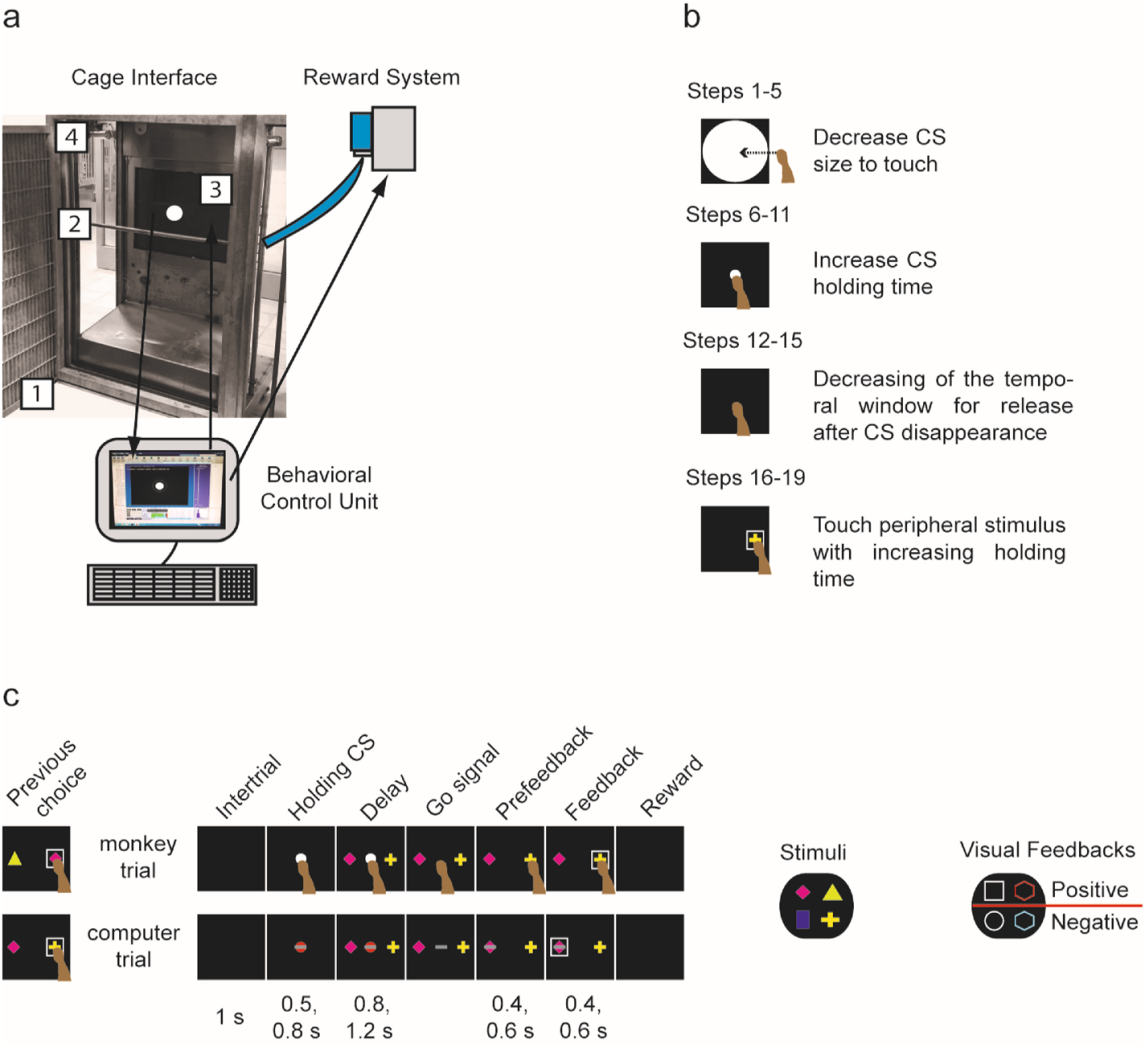
Schematic illustration of the home-cage training system and tasks. (**a**) Home-cage training system. The photograph on the left shows the cage interface (CI) connected through the guillotine door to the experimental cage. The monkey sits in front of the opening of the CI (1) where a stainless steel tube (2) releases the reward via a reward system (top right image). A touchscreen (3) allows the monkey to perform the task. A wide-angle camera (4) monitors the monkey during sessions. The lower image shows the behavioral control unit, which is connected to the touchscreen, reward system, and camera and is used to run the tasks and record the monkey’s responses. (**b**) The 19 steps presented during phase 1 of experiment 1. The monkey learns to touch a central stimulus (CS) of progressively smaller size (steps 1–5); to keep touching the CS while it is on the screen (steps 6–11); to remove its hand after the CS is turned off within a specific time window (steps 12–15), and to touch and continue toughing a target when it is presented on the screen (steps 16–19). (**c**) The sequence of events of one trial of the non-match-to-goal task. The upper section shows a trial performed by the monkeys in phase 2 of experiment 1 and in experiment 2. The lower section shows a trial performed by the ghost agent in experiment 2. The black rectangle represents the touchscreen. The white or red circle is the CS, and the disappearance of the CS represents the go signal for starting the movement. The targets (here, a pink rhombus and a yellow cross) are the stimuli for the choice. The gray rectangle in the computer trial simulates the sequence of actions necessary to carry out a correct complete trial. The first black rectangle on the left shows the target chosen in the previous trial that was, according to the task design, presented again in the current trial, alongside a different stimulus. On the right the four targets and the four types of visual feedback for both experiments are shown. Under the two example trials, the duration of each is shown.

The home-cage training system was developed to allow behavioral training directly in the monkeys’ home cage. It had two components: a cage interface (CI) and a behavioral control unit (BCU) (Fig. 1a). The CI (105 × 55 × 30 cm) was designed to fit against the monkeys’ home cage through the guillotine door as an extension of the cage itself. It was equipped with wheels, allowing it to be moved easily and fixed onto the home cage at the beginning of each session and removed after completion of the session. A stainless steel tube (8 mm inner diameter) placed in the front opening of the frame allowed the release of the reward through a 3 mm hole in its center. The back panel of the CI had an aperture with dimensions suitable for a 17-inch touchscreen monitor (M1700SS 17′′ LCD touch monitor, 1280 × 1024 resolution at 60 Hz, 3 M MicroTouch). The height of the stainless steel reward tube and the touchscreen monitor was adjustable, allowing the CI to be adapted for each monkey. The distance from the monkeys’ eyes to the touchscreen was about 28 cm. During all time spent interacting with the touchscreen, the monkeys placed their mouths as close as possible to the tube to get the reward at the end of a correct trial. A wide-angle camera (ELP 170-degree Fisheye Wide-Angle Camera, 640 × 480 resolution) was attached to the lateral transparent wall of the frame to monitor the monkeys’ behavior during the sessions.

The BCU was located in a separate room, adjacent to the enclosure. It was composed of a desktop computer connected to the touchscreen, the camera, and the reward dispenser. All the behavioral tasks in this experiment and the automated algorithm were written with a MATLAB-based software tool, NIMH MonkeyLogic^46^, allowing the display of the visual stimuli, identification of touches on the touchscreen, real-time monitoring of the monkeys’ behavior during the execution of the task, and recording of the behavioral responses. After each correct trial, water was delivered through the reward dispenser, which consisted of a peristaltic pump connected to the software, allowing the delivery of a precise amount of water.

#### Pre-experiment training and dataset

In the pre-experimental phase, we trained the monkeys to move one by one from any part of the cage to the section where the setup was installed (experimental cage). In this stage, the monkeys were brought into the experimental cage individually for 120 min each, 5 days a week, with free access to 500 ml of water per day for each monkey during this period. The monkeys were rewarded with fruit at the end of this period. After 4 weeks, they moved spontaneously into the experimental cage.

During the experimental phase, for approximately 90 min per day, 5 days per week, we moved one monkey at a time into the experimental cage. A period of 90 min spent in front of the setup on one day was considered a session. We performed two different experiments. In experiment 1, we tested whether the monkeys learned the NMTG task^47^ directly in their home cage, and how this learning occurred. In experiment 2, we tested their ability to perform the NMTG task when alternating with the ghost agent under the same experimental conditions. For both experiments, only correct trials were rewarded, and in the case of an incorrect response, no punishment or timeout was given.

##### Database

We recorded 80 sessions for each monkey (Table 1). We removed two sessions from the data for monkey L and eight from the data for monkey N because in these sessions, a practice version of the task was used in which every trial was always considered correct. We removed another three sessions from the data for monkey N because there were technical problems during those sessions. For both monkeys, the first session of experiment 2 was removed from the data because a practice version of the task was displayed, in which the computer performed the task automatically for the first 10 min. Our analysis categorized each trial, based on its outcome, as correct, incorrect, or aborted.

**Table 1:** Number of sessions used for the analysis for each monkey.

	Experiment 1		Experiment 2	Total
	Phase 1	Phase 2		
Monkey L	4	44	29	77
Monkey N	16	31	21	68

### Ethical statement

The research protocol was approved by the Italian Health Ministry and were conducted according to local and ARRIVE guidelines^48^.

## Experiment 1

### Methods

Experiment 1 consisted of learning the NMTG task, and the training comprised two phases. In phase 1, the monkeys learned, step by step, the basic sequence of actions required in the final version of the task (Fig. 1b), without yet making any choice between peripheral visual stimulus (targets). Visual feedback was displayed at the end of phase 1 as secondary feedback, in addition to the reward. In phase 2, the incorrect target was presented together with the correct target, which allowed the monkeys to begin to learn the NMTG task rule (Fig. 1c). At the beginning of phase 2 of experiment 1, to help the monkeys to understand the NMTG rule, we presented a version of the task in which, at the time of the choice, only the correct target remained on the screen, while the incorrect target was turned off. In this preliminary version it was therefore only possible to make correct choices. This version was presented for two sessions to monkey L and eight sessions to monkey N.

In the NMTG task, the monkeys had to choose between two targets based on the choice previously made, following the non-match-to-goal rule. In one trial (*n*), the touchscreen displayed the correct target from the previous trial (*n* − 1) and a second, different target. The monkeys had to reject the target they had chosen previously and select the other one. There were four possible targets (Fig. 1c), differing in shape and color (blue rectangle, pink rhombus, green triangle, and yellow cross). In cases where an incorrect choice was made (when the monkey chose the same target as in the previous trial), the same pair of targets were presented again, providing a new chance to make the correct choice, until the correct target was chosen. We called these trials “correction trials”. A correction trial could be either correct or incorrect. If it was incorrect, the same trial was repeatedly presented until the correct choice was made and the task could proceed.

### Phase 1

Phase 1 was divided into 19 steps, fully automated and controlled by the following algorithm: to move from one step to the next, the monkey had to have a correct response rate of at least 80% in the previous 10 trials. There were no limitations on the number of steps the monkey could complete in one session. The monkeys could both advance and regress through the steps. In cases where the correct response rate was less than or equal to 20% in the previous 10 trials, the monkey moved back to the previous step, for a maximum of two consecutive steps. In cases where in the previous 10 trials the monkey had not achieved either of the two criteria to change steps, it remained on the same step and another trial was presented using the same step.

- *Steps 1–5:* The goal of the first five steps was to touch a central stimulus (CS) (a white circle) displayed on the touchscreen. Its diameter was decreased gradually from 21 cm in step 1, filling a large part of the touchscreen, to 3.5 cm in step 5. The monkey received the reward when it touched the CS but not the screen outside it. The first three steps were designed primarily to encourage interaction with the touchscreen; for this reason, touching outside the CS was considered an abort and not an error in those steps.
- *Steps 6–11:* From steps 6 to 11, the CS had to be touched and held. In these steps, the duration of the holding time was varied. In step 6 the holding time was set to 500 ms, and this was increased in each subsequent step, to a maximum of 2000 ms in step 10. In step 11, instead, the holding time was not fixed, and its value was determined randomly (1300, 1600, 1700, or 2000 ms). All the trials in which the monkey released its hand before the end of the holding time were considered incorrect and not rewarded.
- *Steps 12–15:* In these steps, the CS had to be touched, held, and then released within a specific temporal window. The temporal release window during step 12 was set to 6000 ms, and this was reduced in each subsequent step until it reached 3000 ms in step 15. Releasing the hand after the duration defined for each step was considered incorrect and not rewarded.
- *Steps 16–19:* In these steps, the CS had to be touched and held for a randomly selected period of 500 or 800 ms. After this period, a target was turned on, randomly selected from the set of four stimuli that would be used in the final version of the task. Then the CS was turned off and the monkey had a maximum of 3500 ms to touch and hold the target. The holding time was increased between each step. From step 17 until the last step, visual feedback (described below) was presented around the target when it was touched, for a step-dependent feedback period.

A trial was considered incorrect if at any time the sequence was interrupted (i.e., the hand was lifted before the end of the holding period). When the actions were performed within the time limit required, and the touch on the target was maintained for the entire duration of the presentation of the visual feedback, a trial was considered correct and rewarded.

### Phase 2

In this phase, the training was not automated. It started with a modified version of the complete task, in which, at the end of the delay period, when the monkey had to make its choice, the incorrect target disappeared, allowing the monkey to choose only the correct one. This version of the task was presented for two sessions to monkey L and for eight sessions to monkey N, based on their interaction with the setup, and these sessions were not included in the data analysis. After these sessions, the monkeys were presented with the complete version of the NMTG task.

In the NMTG task, each trial began with the presentation of the CS at the center of the screen. The monkey had to touch the CS within 5000 ms and hold it for a randomly selected period of 500 or 800 ms. After this period, two targets appeared on the screen, one on the right and the other on the left of the CS, for a randomly selected delay period of 800 or 1200 ms. During this delay period, the monkeys were required to keep holding the CS. After the CS was turned off, the monkey was required to choose and touch one of the two stimuli on the screen within 3500 ms. The monkey then had to hold its hand on the chosen target for 400 or 600 ms (pre-feedback period) until the presentation of the visual feedback. Four types of visual feedback were used, which differed in shape or color (Fig. 1c). Two types of visual feedback were associated with a correct choice (white square and red hexagon) and the other two with an incorrect choice (white circle and blue hexagon). Positive and negative visual feedback types were paired, and the pairs alternated every 20 correct trials. When the correct choice was made, the monkey had to maintain its touch for a randomly selected feedback period of 400 or 600 ms to get the reward. The different types of visual feedback were used because of the need to disentangle the effect of the targets from the outcome in a neurophysiological experiment we have planned for the future.

At the end of both correct and incorrect trials, all targets and visual feedback were turned off, followed by a 1000 ms intertrial interval. Because there was no trial before the first trial of each session, that trial was always rewarded, regardless of which target was chosen.

#### Data and statistical analysis of behavior

During phase 1 of experiment 1, we counted the number of trials needed by the monkeys to move from one step to the next step for the first time. For phase 2 of experiment 1, we calculated the percentage of correct choices in each session. Because each choice was made based on the previous one, we considered only correct and incorrect trials performed after a correct trial. Aborted trials—those in which the monkey either did not interact with the screen or stopped before making a choice—were removed from the analysis, along with trials following aborted trials.

We also analyzed the amount of interaction with the experimental apparatus and the stability of the monkeys’ performance across sessions. We divided the sessions into blocks to assess the degree of commitment to the task. A block was defined as a sequence of at least six consecutive trials. A “working block” started with at least six completed (either correct or incorrect, but not aborted) trials interrupted by no more than five trials in which the monkey did not interact with the touchscreen (non-interactive). A working block ended when there were at least six non-interactive trials performed consecutively. In the same way, a “non-interactive block” started with six consecutive non-interactive trials and ended with six consecutive completed trials; this means that a non-interactive block could include completed trials but no more than six consecutive completed trials.

Next, focusing on the sessions in which the monkeys reached our learning criterion (a correct response rate of 70% in ten consecutive sessions), we calculated the mean percentage of complete and non-interactive trials over the sum of both classes of trial using 100 trial windows, considering the first 800 trials. Sessions with less than 800 trials were removed from the analysis. For each of these sessions, we calculated the number of working blocks the monkeys performed, the number of trials in each of these blocks, and the reaction and movement times of the monkeys in the correct trials in these blocks.

Maintaining a stable performance until the end of the session suggested that the time spent during training was productive, even if the monkey’s interaction with the experimental apparatus became more sporadic. To assess the stability of the performance, we compared the two halves of the sessions that had reached the learning criterion described above, considering both correct and incorrect trials (Wilcoxon signed-rank test). Each half session comprised 50% of the trials.

## Results

**Figure 2:**
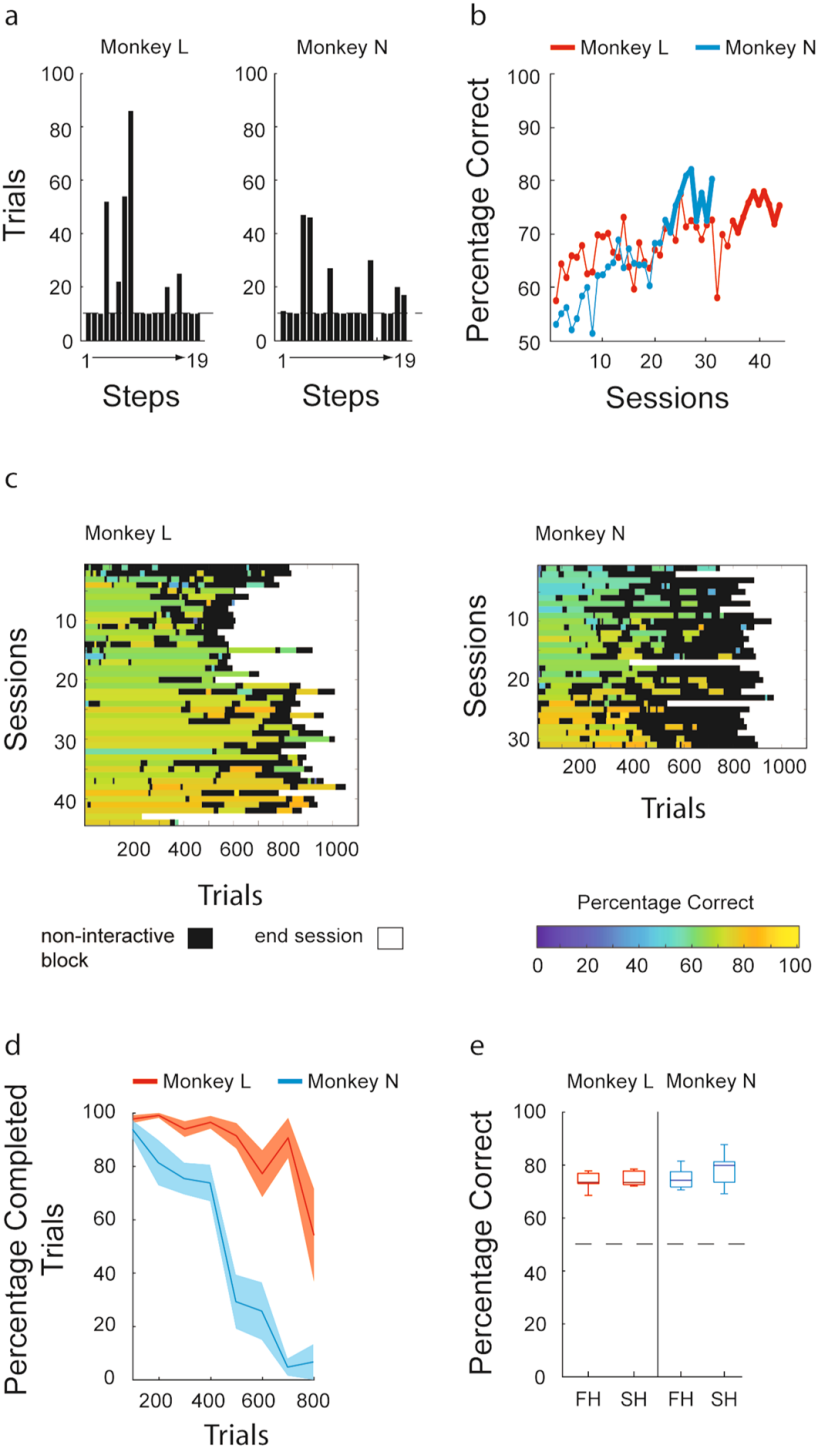
Behavioral results of experiment 1. (**a**) Results of phase 1 and correct response rate of phase 2. Bars represent the number of trials performed to complete a step that was presented for the first time during phase 1. Dashed lines indicate the minimum number of trials required to reach the next step. (**b**) Learning curves for both monkeys. The curves show the percentage of correct choices for each session during phase 2 (44 sessions for monkey L and 31 for monkey N). The thick lines represent the 10 consecutive sessions in which the monkeys reached a correct response rate of above 70% (where a correct response rate of 50% corresponds to chance). (**c**) Working blocks. Each row represents a session during phase 2. Colored lines indicate working blocks, which comprise completed trials (see “Experimental procedures” section). The color bar indicates the correct response rate calculated for each working block. Black lines indicate the non-interactive blocks, which comprise trials in which the monkeys did not interact with the touchscreen. The white area indicates the end of the sessions. (**d**) Stability of interaction within phase 2 sessions. The curves indicate the mean percentage of complete trials (see experimental procedures) calculated using a moving window of 100 trials in sessions in which the learning criterion was achieved and which included at least 800 trials. Color-shaded regions represent the standard error of the mean. (**e**) Stability of performance within phase 2 sessions. Correct response rate for sessions in which the learning criterion was achieved and which included at least 800 trials. The sessions are split into two halves: first half (FH) and second half (SH). The central line represents the median and the top and bottom borders of each box represent the 25th (p25) and 75th (p75) percentiles of the two groups of trials. The whiskers represent the range from 1.5 times the interquartile range above p75 (p75 + 1.5 [p75 − p25]) and below p25 (p25 − 1.5 [p75 − p25]). Data points outside this range are plotted individually (red crosses). The dashed line indicates 50%, the level associated with responses dictated by chance alone.

During phase 1 of experiment 1, monkey L performed 737 trials in four sessions and monkey N performed 1819 trials in 16 sessions, resulting in a mean of 184 trials per session and 122 trials/h for monkey L and a mean of 113 trials per session and 88 trials/h for monkey N. The criterion of 80% correct in the first 10 trials of a step was reached by monkey L and N in 13 and 10 steps, respectively, out of the total 19 steps (Fig. 2a). Both monkeys needed more trials to calibrate their touch responses to the final size of the CS in step 4. In addition, monkey L needed more trials to learn to hold the CS for a longer time, which corresponded to steps 7 and 8.

Monkey N successfully completed the steps requiring touching (steps 1–5) and holding the CS (steps 6–11) and the target (steps 16–19). However, monkey N was unable to perform step 15, in which it was required to remove its hand from the touchscreen after the disappearance of the CS and before the targets were presented. This difficulty affected the monkey’s motivation to work in the sessions involving step 15. We thus decided to restart the training from the first step with the purpose of restoring its motivation to interact with the setup. Taking into account the difficulty this monkey had with step 15, we decided to remove steps 12–15, because in these steps the monkey had to perform trials based on the rule that it was unable to learn (to remove its hand from the touchscreen). After this restart, the monkey had no difficulty progressing to step 16, i.e., it jumped from step 11 to step 16.

We then moved to phase 2, in which the monkeys were trained until they reached a stable performance, achieving a correct response rate of above 70% for 10 consecutive sessions. Both monkeys learned the NMTG rule, progressively increasing their correct response rates and achieving the criterion in 44 and 31 sessions for monkeys L and N respectively, with an average correct response rate of 74.68% and 76.31% during their last 10 sessions (Fig. 2b).

Monkey L performed, on average, 517 completed trials per session, making a total of 22,753 completed trials. Monkey N performed, on average, 362 completed trials per session, making a total of 11,278 completed trials. Figure 2c shows an overview of the monkeys’ involvement and performance over time within the sessions of phase 2, based on the trials in the two types of behavioral block (working and non-interactive; see “Experimental procedures” section).

We focused our subsequent analyses on obtaining quantitative measures of the stability of the monkeys’ performance and interaction with the experimental apparatus. We did this because we wanted to evaluate the risk that excessively long sessions could have a negative effect on these two behavioral parameters. In both cases, we considered only the last 10 sessions collected after the monkeys had learned the task. Following a criterion that the analysis had to be based on a minimum of 800 trials in a session (completed plus non-interactive trials), we removed three sessions from the data for monkey L and one from that for monkey N. The monkeys exhibited a consistent duration of interaction with the experimental apparatus, interspersed with a limited number of interruptions, maintaining an average of over 80% complete trials for 500 and 200 trials for monkey L and N, respectively, after a session began (Fig. 2d). They performed on average 3.50 (standard error of the mean [SEM]: ± 0.58) and 4.62 (SEM: ± 0.33) working blocks, respectively, consisting of 143.80 (SEM: ± 25.67) and 68.50 (SEM: ± 9.32) complete trials (Supplementary Fig. S1a). The average reaction times were 411.75 ms (SEM: ± 3.08) and 396.65 ms (SEM: ± 5.02) respectively for monkey L and monkey N, and movement times were 293.59 ms (SEM: ± 0.90) and 226.32 ms (SEM: ± 0.90) respectively for monkey L and monkey N.

We tested the stability of the monkey’s performance (Fig. 2e) during the last 10 sessions with a minimum of 800 trials. We did not find any significant difference for either monkey between the average percentage of correct trials during the first and the second halves of the sessions (monkey L: 74.00% vs 74.99%, *V* = 50, p = 0.8048; monkey N: 74.92% vs 77.92%, *V* = 71, p = 0.2224).

## Experiment 2

### Methods

In experiment 2, we first wanted to assess whether the monkeys could extract and take into account the information from the choice made by the ghost agent to make their choice correctly in the next trial. We then analyzed the stability of the monkeys’ performance and interaction with the experimental apparatus, as described previously, for this version of the task.

In this version of the NMTG task, the trials in which the monkey observed the ghost agent completing the trial were interspersed with trials performed by the monkey, according to a rule described below. In this way, we could distinguish two different classes of trials: those performed by the monkey (monkey trials) and those performed by the ghost agent (computer trials) (Fig. 1c).

The monkey trials were the same as those described for the standard version of the NMTG task: the monkey was required to choose the target according to the NMTG rule, depending on its own previous choice if the previous trial was a monkey trial, or on the ghost agent’s choice if the previous trial was a computer trial. However, the computer trials had some differences. A computer trial started when a red CS appeared, instead of a white CS as in the monkey trials. In the first 10 min of the first session, the monkeys learned to recognize a red CS and to wait without touching the screen for the duration of the trial when it was presented. The temporal sequence of the events of a computer trial was identical to that of a monkey trial except that a gray rectangle mimicked the performance of the different phases of the trial. After the red CS appeared, the gray rectangle appeared over the CS for a randomly selected period of 400 or 600 ms. Next, two targets appeared and after a delay period of 800 or 1200 ms, the gray rectangle moved horizontally at a constant speed of 0.11 m/s, reaching the correct target within 1000 ms. After 400 or 600 ms of the pre-feedback period, the visual feedback for a correct trial was displayed. In these trials, the ghost agent automatically chose the correct target based on the choice made in the trial before (either a monkey or a computer trial). If the monkey had not touched the touchscreen during the whole trial, it received the reward at the end. Alternatively, if it touched the touchscreen at any time during a computer trial, the trial was aborted, and the same trial was repeated until it had been completed.

A session was composed of 1–4 correct monkey trials interspersed with 1–4 computer trials. Varying the number and order of computer and monkey trials prevented the monkeys from predicting the agent in the next trial, which kept the monkeys focused, even on computer trials that were followed by other computer trials. A computer trial could only start after a correct trial. During the monkey trials, if the incorrect choice was made or the trial was aborted, that trial was not included in the count.

#### Data and statistical analysis of behavior

We evaluated the percentage of correct choices during the whole session to assess the ability of the monkeys to monitor the ghost agent’s choices during the computer trials and then to continue the task in the monkey trials. As in experiment 1, we calculated the average percentage of complete (correct, error, and computer) and non-interactive trials in 100 trial windows, using all sessions. Since the monkeys had already been trained in experiment 1 and were able to perform the task, we wanted to evaluate whether, despite the new condition, they continued to demonstrate an ability to maintain a stable performance during the sessions. We also wanted to test, first, whether (as expected) the monkeys would still demonstrate a good and stable performance following a monkey trial, as was observed in experiment 1. Second, we wanted to evaluate their performance after computer trials. We separated the trials into two categories based on which actor had performed the previous trial: “after-monkey trials” (the monkey performed the previous trial) and “after-computer trials” (the ghost agent performed the previous trial).

We evaluated the monkeys’ performance in these two trial categories. The percentage of correct trials in each category was calculated as the ratio of correct trials to the sum of all trials (correct and incorrect) in that category. To evaluate the stability of the monkeys’ performance within sessions, we divided the sessions into two halves, as for experiment 1. We considered the two trial categories separately and then performed a Wilcoxon signed-rank test to compare the first and second halves of all sessions.

We also calculated the percentage of trials aborted after computer trials. A low percentage of aborted trials would indicate that the monkeys had been monitoring the computer trials and immediately resumed working when it was their turn again. Finally, as for phase 2 of experiment 1, we calculated the number of working blocks for each session. For each working block we calculated the number of trials executed, including computer trials, and the performance of the monkeys. Reaction and movement times were calculated based on the correct trials performed by the monkeys in these blocks.

## Results

**Figure 3:**
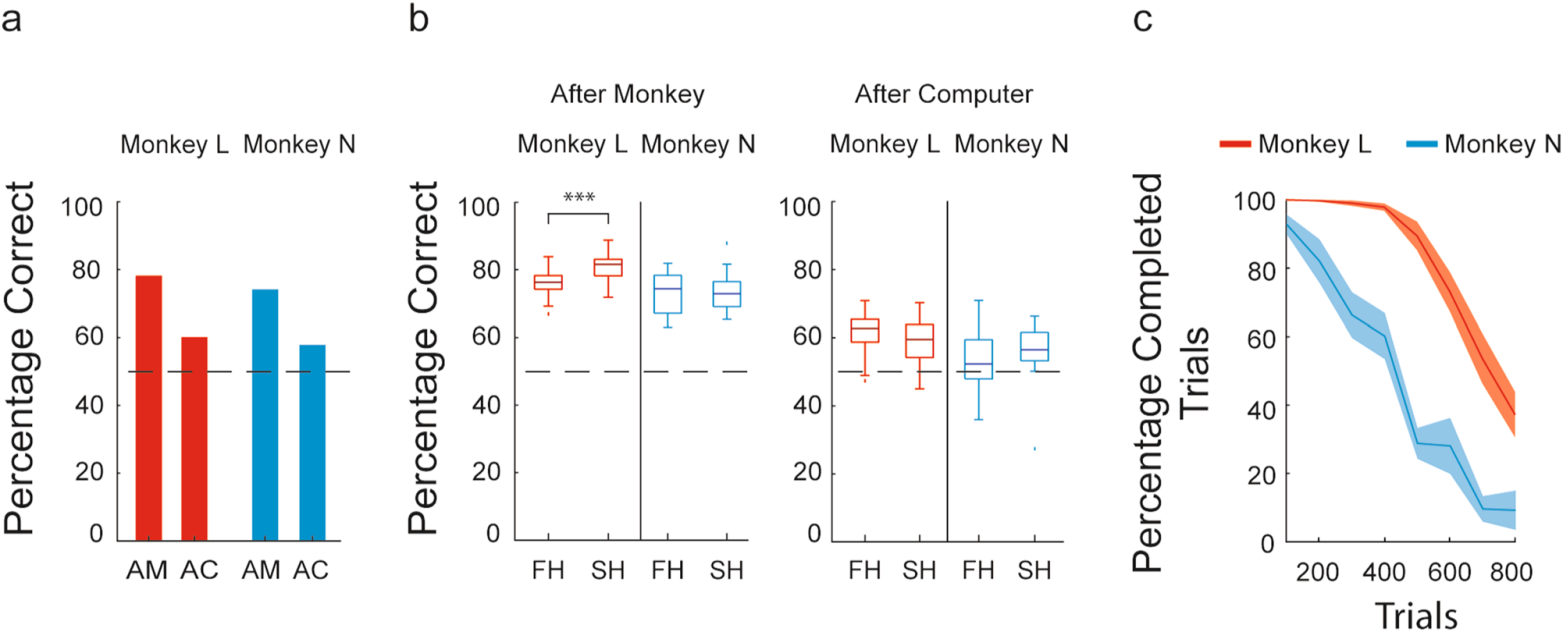
Behavioral results of experiment 2. (**a**) Overall performance. Bars represent the percentage of correct choices in after-monkey and after-computer trials, based on all the sessions in experiment 2 (29 and 21 sessions for monkeys L and N, respectively). (**b**) Stability of the monkeys’ performance within sessions in after-monkey and after-computer trials. The structure of the boxplots and calculation of the percentages of correct choices are as described in Fig. 2e, except they apply to the after-monkey and after-computer trial categories. In both graphs, the dashed line indicates 50%, the level associated with responses dictated by chance alone. Asterisks indicate significant differences (***p < 0.001, Wilcoxon signed-rank test). (**c**) Stability of the monkeys’ interaction with the apparatus. The curves represent the mean percentage of completed trials (see experimental procedures) calculated in moving windows of 100 trials, using the sessions presented in (**a**).

In experiment 2, monkeys L and N performed 11,167 and 4774 trials (monkey trials) and monitored 7482 and 3159 trials completed by the ghost agent (computer trials), respectively, with a mean of 643 per session and 428 trials/h for monkey L and a mean of 377 per session and 251 trials/h for monkey N. We first assessed whether the monkeys were able to make correct choices based on the choice made previously by the ghost agent. The mean correct response rate in after-monkey trials was 78.25% for monkey L and 74.24% for monkey N. In the after-computer trials, however, their correct response rates were 60.24% and 57.95%, respectively. Although their performance was weaker in after-computer trials, both monkeys still maintained a performance better than chance in both the after-monkey trials (exact binomial test: both monkeys: p < 2.2e^−16^) and the after-computer trials (exact binomial test: monkey L: p < 2.2e^−16^; monkey N: p = 1.5e^−07^) (Fig. 3a).

As in experiment 1, we evaluated the stability of the monkeys’ performance and of their interaction with the experimental apparatus within the sessions of experiment 2. Monkey L and monkey N, for 500 and 200 trials, respectively, exhibited a low interruption rate and an average percentage of completed trials, from the start of the session, of above 80% (Fig. 3b). With respect to the stability of their performance, in after-monkey trials, monkey L exhibited a better performance in the second half of the sessions (75.97% vs 80.50%, *V* = 23, p = 4.1e^−05^), while the performance of monkey N appeared to be stable (73.17% vs 73.66%, *V* = 65, p = 0.5862). In the comparison of after-computer trials between the first and second half sessions, we found no significant difference for either monkey (monkey L: 61.36% vs 58.83%, *V* = 270, p = 0.087; monkey N: 55.62% vs 58.54%, *V* = 40, p = 0.27) (Fig. 3c).

Respectively, monkeys L and N completed on average 3.03 (SEM: ± 0.16) and 4.61 (SEM: ± 0.24) working blocks (Supplementary Fig. S2), consisting of 151.01 (SEM: ± 24.89) and 62.79 (SEM: ± 8.02) complete trials (Supplementary Fig. S1b). Their average reaction times were 486.35 ms (SEM: ± 2.72) and 398.82 ms (SEM: ± 4.77), respectively, for monkey L and monkey N, and movement time were 303.61 ms (SEM: ± 0.90) and 228.99 ms (SEM: ± 0.81) respectively, for monkey L and monkey N. Finally, monkeys L and N aborted only 3.05% (SEM: ± 0.39) and 6.15% (SEM: ± 0.76) of the after-computer trials, respectively.

## Discussion

In this study, we evaluated the ability of two macaque monkeys to be trained in their home cage in two experiments. We report three main findings. First, we showed that both monkeys were able to learn a high cognitive demanding task in their home cage and, in the first and second experiments, respectively, performed alone and in interaction with a ghost agent. Second, they maintained their involvement in the task in terms of the percentage of completed trials, with few interruptions, across sessions for a large number of trials (200–500, depending on the individual). Third, in the second halves of the sessions, even if there was reduced interaction with the apparatus, the monkeys’ performance was maintained.

In the initial phase of the home-cage training, we based the training in phase 1 of experiment 1 on the method described by Berger et al.^34^, but allowed greater flexibility. In fact, we decided to introduce some changes when we thought we could enhance the learning process. Indeed, some steps appeared more challenging to complete than others, such as step 15, and to avoid long delays in learning the task we intervened and altered the rules defined by the algorithm. In our protocol, the numbers of steps planned for the initial training and of trials necessary to reach the next step were set lower than in the protocol used by Berger et al.^34^.

Since monkeys exhibit individual differences^49^ and different learning speeds, the automatic training control algorithm made it possible to calibrate the training to each monkey’s abilities. In phase 1 of experiment 1, monkey L learned faster than monkey N. Monkey N showed difficulties in step 15 because, in the absence of a stimulus after the “go-signal”, it was unable to understand that it had to take its hand off the touchscreen. We encountered the same difficulty that Berger et al.^34^ found in a similar step of their protocol with the same requirements (removing the hand from the touchscreen, step 30 in the Berger protocol), and we therefore intervened directly, with the intention of facilitating the continuation of the training. We decided to use an approach that was not completely controlled by the algorithm, since the aim of this study was not to validate a standardized tool or to characterize individual differences^34^. For these reasons, we decided to remove the steps that required the monkey to take its hand off the screen, allowing monkey N to complete the training. Consequently, our study supports the importance of using an algorithm to enhance learning but also raises a concern about the use of an inflexible, fully automatized process. We believe that to allow the monkeys to overcome some critical steps, the experimenter should be able to act flexibly and intervene when needed, to make the appropriate changes to the training protocol.

We must also consider, however, that during this first phase, the monkey’s background and former training may have facilitated its approach to the touchscreen, since both monkeys were already aware that if they completed a trial correctly, they would receive a reward. Taking this into account, the use of completely naive monkeys may require more effort and changes to the approach used in the initial phase of training. We believe that increasing the number of sessions and the number of trials needed to complete a step may help the monkeys to learn and refine correct responses and reinforce the action–reward association. A semi-automated approach may be particularly beneficial in this context, allowing training to be adapted to the different learning speeds of individual monkeys by splitting or changing the learning steps.

The NMTG task is considered to be a task with a high cognitive demand, because the monkeys are required to apply a counterintuitive rule^47,50–54^ and continuously monitor the trials performed, because the correct choice in one trial depends on the previous trial. We showed that this task could be learned using a home-cage training approach. Previous studies have investigated the capacity of monkeys to learn complex tasks under similar task conditions^8,9,38–41,55^. Hutsell and Banks^55^ utilized a delayed non-match-to-sample paradigm to study the effect of environmental and pharmacological manipulations on working memory. However, unlike our paradigm, in their task the correct choice did not depend on knowledge of what happened in the previous trial.

As in other studies^36,42^, one of our goals was to evaluate the stability of performance, but we did this within and not between sessions. This approach allowed us to investigate how the monkeys interacted over time with the experimental apparatus within sessions and the time allowed—a practice commonly used in neurophysiology studies. Our results show that both monkeys started interacting at the beginning of the session and continued in a constant and continuous way for a substantial number of trials (500 for monkey L and 200 for monkey N). In terms of the number of trials performed, our results are comparable with those reported by a previous study conducted using a similar task but in an experimental setup where the monkeys worked in a primate chair^50^.

The monkeys’ involvement in the task progressively became more sporadic, with alternating phases of interaction and non-interaction, presumably due to a progressive reduction in interest in the reward or because of the attentional effort required by the task. These problems are also common in laboratory training. Considering the stability of their performance during each session, however, our results are very encouraging, and suggest that regardless of the sporadic nature of the monkeys’ behavior in the latter part of the sessions, they were still able to implement the task rules correctly and the training time was still effective.

In experiment 2, we demonstrated that both monkeys were able to monitor the choices of the ghost agent, maintaining a stable performance not only in the trials following their own choices, but also in those following trials completed by the computer. Some previous studies have investigated social interactions using computers as nonsocial agents^45,56^. Here, in addition to confirming that macaque monkeys are able to extract information from a ghost agent, we assessed their ability to maintain their involvement in task execution and to interact dynamically with the ghost agent in their housing environment. However, it is important to note that the correct response rate of monkey N (57.95%) was only slightly better than that dictated by chance (even though this difference was statistically significant). Further comparison of the performance of the same monkey when in a primate chair may help us to understand whether its difficulty applying the NMTG rule during home-cage training was due to a lack of focus specific to the home-cage environment.

Subiaul et al.^57^ used the ghost display condition to study observational learning. In their cognitive imitation study, two monkeys had to observe a conspecific or ghost agent selecting a series of images presented on a screen according to a specific sequence, and were subsequently tested on the same set of images. Observational learning occurred after observation of the conspecific only, and not after observation of the ghost agent, indicating that the monkeys were unable to extract information from the ghost agent, at least in that experimental setup. Unlike Subiaul et al.^57^, we found that our monkeys were able to extract information from the ghost agent, as indicated by the fact that their correct response rates were above 50% in the after-computer trials. These correct response rates and the low rate of aborted trials in after-computer trials together indicate that the monkeys were able to remain focused for most of the session. This discrepancy between studies (in terms of information extraction from the host agent) may be accounted for by the delivery of a reward to the monkey, which did not occur in the study of Subiaul et al.^57^, as previously suggested by Ferrucci et al.^44^.

At the end of both experiments, monkey N had performed fewer trials than monkey L. The variability we observed within our monkey sample was similar to that observed by Berger et al.^34^. In addition, however, as in our study, they reported that the number of interactions with the setup did not affect the monkeys’ performance.

Combined, these results indicate that a home-cage setup could represent a valuable tool for teaching even complex tasks that require a great deal of cognitive and attentional effort throughout the session. We found that even in the context of the home environment, where each monkey could maintain visual contact with its cage-mate^58^, the monkeys were able to maintain the necessary attention on the task requirements and achieve a good performance and an adequate number of trials. Previous studies that have demonstrated the possibility of learning cognitive tasks directly in the home cage, together with the development of new technologies, are introducing the possibility of studying cognitive and behavioral processes in more natural contexts. Furthermore, as observed by Gazes et al.^59^, monkeys that lived in large social groups and had learned to perform tasks directly in their environment were as productive for research as laboratory-housed monkeys. Home-cage training thus represents a useful tool in neuroscience experiments for teaching monkeys to perform tasks ahead of neural recordings taken in the laboratory.

Different cognitive functions investigated in neurophysiological experiments require pre-training to execute more than just single trials in isolation, although this is rarely mentioned. For example, different behavioral tasks used to study the generation of goal-directed rules and responses^60,61^, logical reasoning^62^, mapping of stimulus–response associations^63^, and one-trial learning^44,64^ require monkeys to constantly take into account various task variables learned in previous trials and carry out subsequent trials during the session, so that the neural correlates involved can be studied. In addition, a common approach in electrophysiology consists of requiring the monkey to repeat the same behavior several times and recording the neural activity during this behavior. Given these requirements, achieving a constant level of behavioral involvement during sessions, a low level of variability between and within sessions, and stable performance can be considered key objectives in neurophysiology studies for both the initial training and the collection of neural data.

Thanks to technological advances and the development of wireless recording devices^65–68^, home-cage training will not only be used to teach tasks before initiating recordings in neurophysiology experiments in which the monkeys would still need to be moved to an experimental room, but could also completely change the laboratory setting, since the whole experiment could be performed without removing the monkeys from their home cage. Despite the potential advantages of this approach, some important technical problems remain to be addressed to develop the necessary controls for motor behavior. Such controls would be a prerequisite for the interpretation of neural data recorded in this naturalistic setup. In cases where the neural recording will still be performed in the laboratory, it will be important to investigate whether the learning obtained in the home cage is easily translated to the laboratory setup.

## Supplementary Information

Supplementary Information.

## Data Availability

The dataset generated and/or analyzed during the current study is available from the corresponding author on reasonable request.
