# Supplementary material for: Macaque monkeys learn and perform a non-match-to-goal task using an automated home cage training procedure
Source: Sci Rep. 2021 Jan 29;11:2700. doi: 10.1038/s41598-021-82021-w (PMC7846587; doi:10.1038/s41598-021-82021-w)
Supplement: Supplementary file 1 — Supplementary Information. [file 41598_2021_82021_MOESM1_ESM.pdf]

Title: Macaque monkeys learn and perform a non-match-to-goal task using an automated home cage training procedure

Authors name and affiliation: Stefano Sacchetti[1,2,3], Francesco Ceccarelli[1,2,3], Lorenzo Ferrucci[1], Danilo Benozzo[1], Emiliano Brunamonti[1], Simon Nougaret[1]*, Aldo Genovesio[1]*.

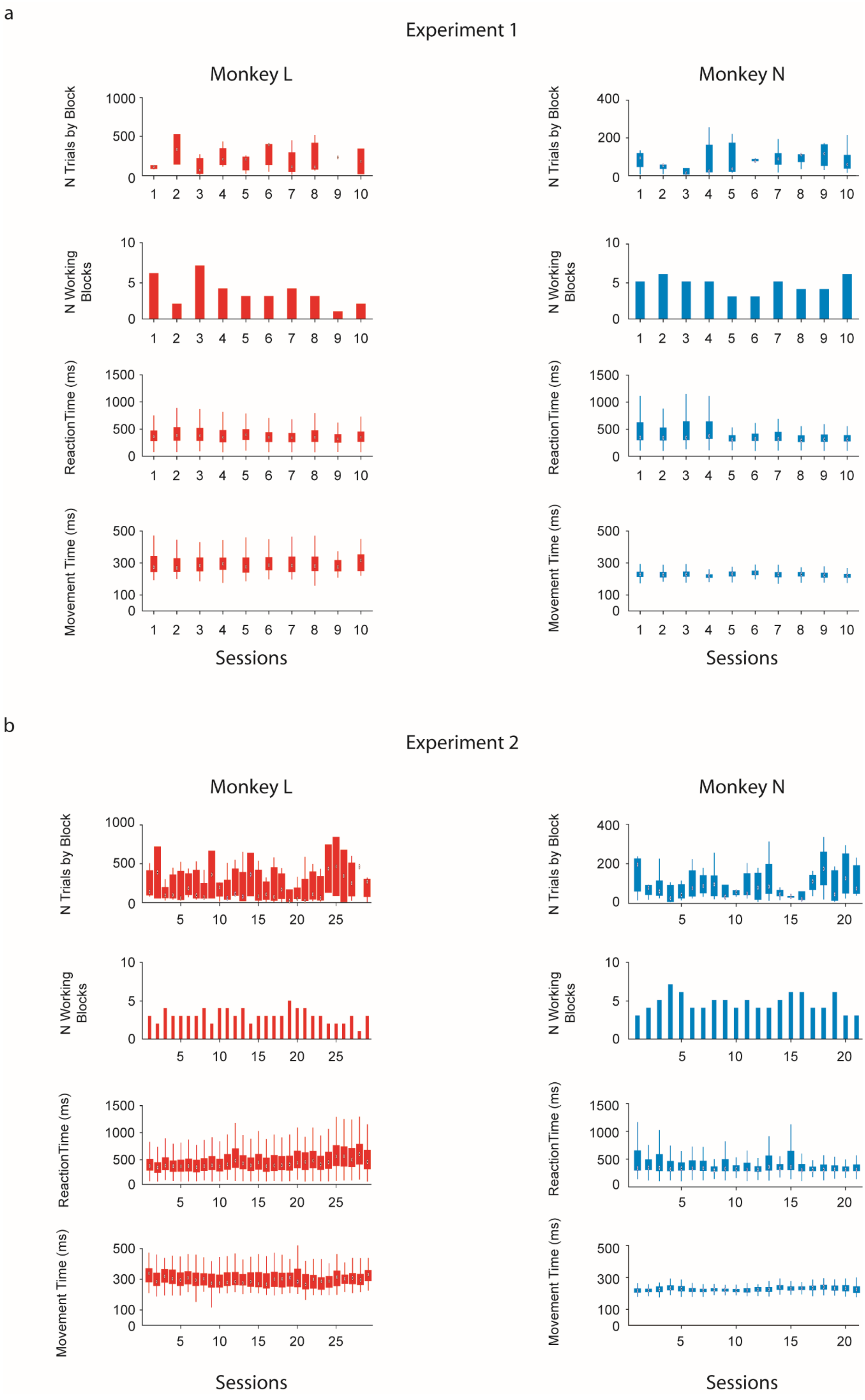

a
Experiment 1
Monkey L
Monkey N
N Trials by Block
N Working Blocks
ReactionTime (ms)
Movement Time (ms)
Sessions
b
Experiment 2
Monkey L
Monkey N
N Trials by Block
N Working Blocks
ReactionTime (ms)
Movement Time (ms)
Sessions

S1. Behavioral parameters of both experiments. (**a**) Additional information about the monkeys' task involvement during experiment 1. From top to bottom: number of trials in each working block; number of working blocks in each session; reaction times in each session; and movement times in each session. The x-axis of each plot represents single sessions. Note that the Y axis of the first boxplot is different between monkeys. (**b**) Additional information about stability during experiment 2. From top to bottom: number of trials in each working block; number of working blocks in each session; reaction times in each session; and movement times in each session. The *x*-axis of each plot represents single sessions.

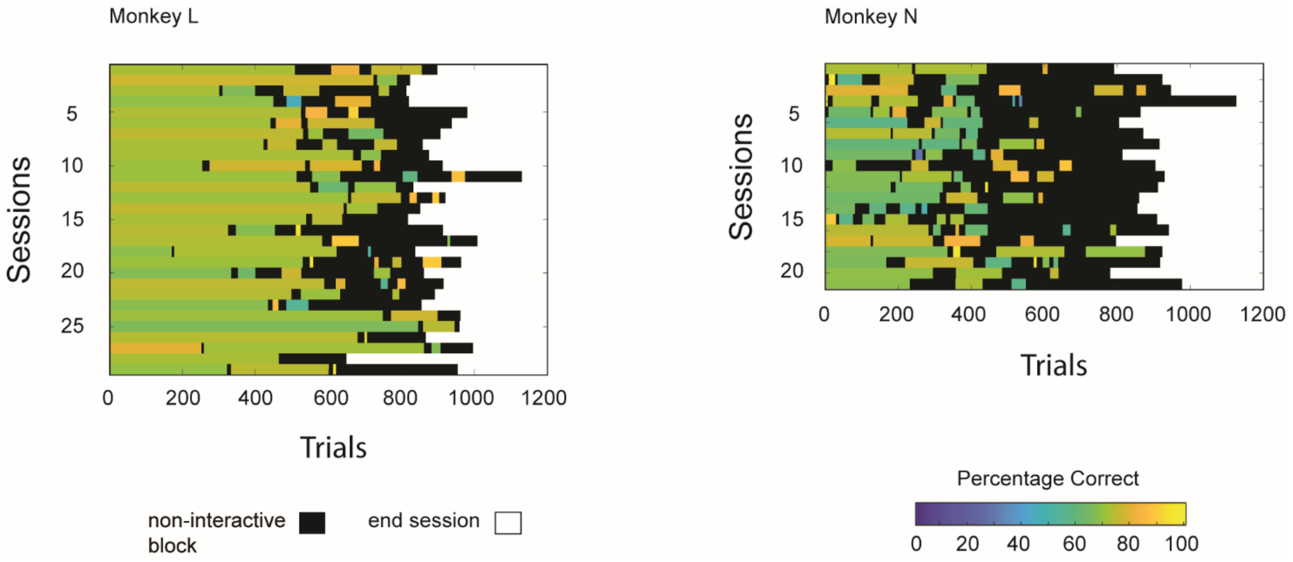

S2. Working blocks in experiment 2. Each row represents a session. Colored lines indicate working blocks composed of monkey trials and computer trials (see experimental procedures). The color bar represents the percentage of correct choices calculated for each working block. Black lines indicate non-interactive blocks, composed of trials in which the monkeys did not interact with the touchscreen. The white area indicates the end of the session. The performance within the working blocks is calculated based only on monkey trials.
